# Evaluation of Two Mini-implant Lengths in the Infrazygomatic Crest Region: A Randomized Clinical Trial

**DOI:** 10.1055/s-0044-1789015

**Published:** 2024-11-07

**Authors:** Abbas F. Alsaeedi, Mehdi Abdul-hadi Alrubayee, Gautham Sivamurthy

**Affiliations:** 1 Department of Orthodontics, College of Dentistry, University of Baghdad, Baghdad, Iraq; 2 Department of Orthodontics, School of Dentistry, University of Dundee, Dundee, United Kingdom

**Keywords:** miniscrews, infrazygomatic crest area, TADs, orthodontics, mini-implant

## Abstract

**Objective** Temporary anchorage devices (TADs) have revolutionized fixed orthodontic appliance treatment through anchorage controlling in the clinic and play an essential role in resolving many complex cases. Due to the limited space, there is a risk of injury to the roots while using interradicular microimplants. Therefore, the infrazygomatic crest (IZC) area can be an alternative mini-implant insertion site in the maxillary arch. The aim of the study was to evaluate the primary stability, pain perception, sinus penetration, late stability, and failure rate with two mini-implant lengths in the IZC area.

**Materials and Methods** Forty-eight mini-implants (Tusk Dental Co., Ltd., Canada) with two different lengths (length/diameter: 12/2 and 14/2 mm) were grouped by length (24 per group) and inserted bilaterally into the IZC area of 24 patients. The data were statistically analyzed, considering a significance level of *p* < 0.05.

**Results** Sinus penetration prevalence did not differ significantly between 12-mm (54.2%) and 14-mm (62.5%) mini-implants ( *p* > 0.05). Primary stability was significantly higher with the 14-mm mini-implants ( *p* < 0.05). The failure rate did not differ significantly between the 12-mm (20.8%) and 14-mm (16.7%) mini-implants ( *p* > 0.05).

**Conclusion** While the failure rate was similar between 12- and 14-mm mini-implants, the 14-mm mini-implants were more likely to damage adjacent structures. Therefore, shorter mini-implants should be preferred over longer mini-implants for most cases requiring IZC TADs.

**Trial Registration ID** ClinicalTrials.gov identifier: NCT06293872.

## Introduction

Orthodontic treatment mainly depends on two mechanics: facilitating the desired teeth movement to the new position and preventing unwanted teeth movement, which requires anchorage control. Anchorage enhancement has progressed greatly over the past century, and one of these milestones was the development of mini-implants placed intraradicularly. Recently introduced extraradicular mini-implants, including temporary anchorage devices (TADs), are inserted into the infrazygomatic crest (IZC) area in the upper arch and buccal shelf (BS) in the lower arch. 1 Intraradicular and extraradicular mini-implants have accompanied a renaissance in orthodontics over the last decade, introducing the concept of absolute or maximum anchorage, in addition to anchorage recently used to accelerate tooth movement. 2 These advancements serve as additional tools for orthodontists, enabling them to address new clinical challenges and transform borderline surgical cases into nonsurgical ones without compromising the achieved results. 3

Intraradicular TADs have many limitations, such as root proximity, which carries the risk of root damage, a major risk factor for TAD failure. 4 Their placement between roots may also restrict the full arch movement as it interferes with mesiodistal root movements. 5 To reduce eventual failures due to proximity to the roots and enable orthodontic mechanics to obtain adequate tooth movements, orthodontists have attempted to insert TADs into extra-alveolar regions such as the IZC and BS. 6 Studies have shown that from a clinical perspective, the IZC TAD position remains stable and could be efficiently used for anchorage to improve orthodontic tooth movement. 7

Furthermore, inserting TADs into the IZC has many advantages, such as thicker bone, which allows the insertion of longer mini-implants, greater bone contact, and better primary stability. 8 In addition, the two cortical plates (the buccal cortical plate and the sinus floor) have greater bone density (an anatomic advantage), which may provide better primary stability for the mini-implant due to bicortical fixation. Moreover, TADs inserted into the IZC have another advantage over intraradicular mini-implants: they allow full arch distalization without root contact issues. 9

Maxillary sinus perforation is considered a major issue when using TADs inserted into the IZC. 10 However, Chang et al reported that it does not affect the 6-month postinsertion survival rate, and thus, the failure rate of TADs inserted into the IZC. 10 Nonetheless, sinus penetration is still regarded as a vital structure damage. Moreover, evidence suggests that involving the sinus to enhance primary stability is unnecessary, where larger TADs compromise bone integrity over a greater area than smaller TADs; therefore, larger TADs should be avoided when possible.

Additionally, the optimal combination of mini-implant size and insertion angle is critical for achieving good primary stability, reducing the risk of sinus penetration, and having high clinical performance. 11 While failure rates may appear no better with longer mini-implants than with shorter mini-implants, they do have disadvantages regarding potential side effects. A longer implant has a higher likelihood of damaging adjacent structures. 12 Therefore, this study examined how the length of the TAD inserted into the IZC affects sinus penetration, stability, and failure rate, which have been insufficiently explored in the previous studies. 10 It compares the two longest available IZC mini-implants (12 and 14 mm) used in the IZC area. 13

## Aim of the Study

This study aimed to examine how the length of the TADs inserted into the IZC affects sinus penetration, stability, and failure rate.

## Objectives

### Primary Objective

The primary objective was to evaluate the influence of mini-implant length on its early stability, long-term stability, and failure rate in the IZC area.

### Secondary Objectives

The secondary objective was to assess how mini-implant length affects sinus penetration and patients' pain perception 1 week postinsertion.

## Materials and Methods

### Study Design

This study was a single-operator, split-mouth, double-blind, randomized clinical trial with a 1:1 allocation ratio and per protocol analysis.

### Subjects/Settings

Forty-eight patients received TADs inserted bilaterally into the IZC. This study was ethically approved by the ethics committee of the College of Dentistry, University of Baghdad a before it commenced (project no. 784423) and registered at ClinicalTrials.gov (ID: NCT06293872). Patients who met the inclusion criteria were asked to assign a comprehensive consent form before the start of the study.

## Procedure/Intervention

### Sample Selection

Consecutive patients were eligible to participate in this study if they met the following inclusion criteria: (1) aged 18 to 30 years, (2) patients currently receiving orthodontic treatment with the use of a fixed orthodontic appliance and need mini-implant placement into the upper buccal posterior area (IZC), (3) patients who have the desire and ability to comply with the trial protocol, and (4) recommended for the use of bilateral miniscrews. The exclusion criteria were as follows: (1) clinical examination suggesting sinus inflammation or a history of chronic sinusitis or sinus surgery pathology before mini-implant insertion, and (2) syndromic disease, facial trauma, and/or a history of surgery for bone disease. The dropout criteria were as follows: (1) postinsertion cone beam computed tomography (CBCT) showed that the TAD was inserted interradicular rather than into the IZC area, and (2) the patient decided to withdraw from the study.

### Randomization

**Fig. 1:**
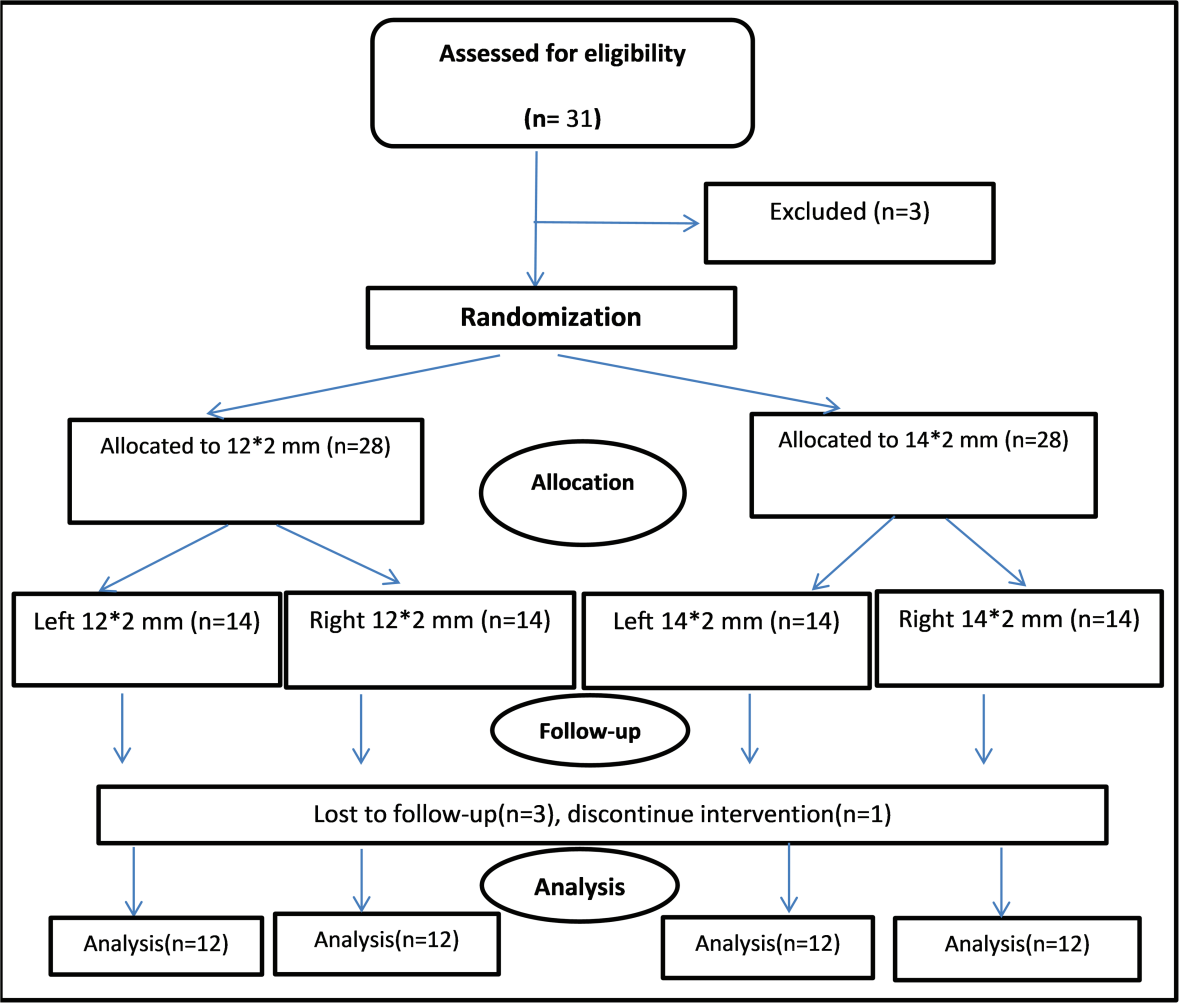
Flowchart illustrates the workflow of the randomized clinical trial.

According to a randomized split-mouth design ( Fig. 1 ), each patient received a 12-mm mini-implant on one side and a 14-mm mini-implant on the other side. 13 The mini-implant pairs were coded for the right and left sides and arranged in an alternating form to guarantee that an equal number of each length was inserted into the right and left sides. 14

### Surgical Procedure

**Fig. 2:**
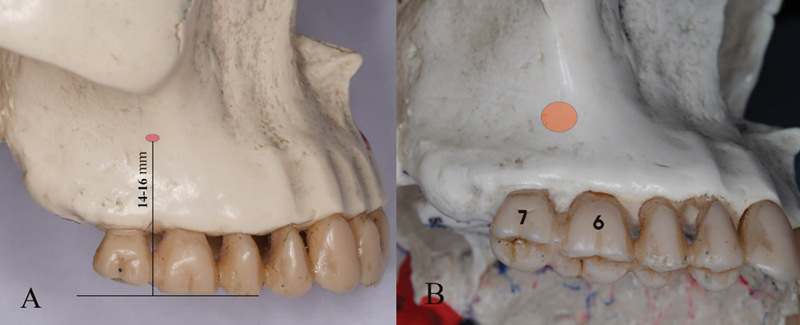
Insertion site of mini-implant. ( **A** ) Vertical distance for infrazygomatic crest (IZC) temporary anchorage device (TAD) from the occlusal surface of upper first molar and ( **B** ) insertion site (orange) for IZC TAD.

Before mini-implant insertion, the patients were instructed to rinse their mouths with chlorhexidine mouthwash, and then local anesthesia was applied. The mesiodistal site for mini-implant insertion located between the first and second molars is the most frequently recommended insertion site for TADs in the IZC. The mini-implants were placed by a postgraduate student (A.A.) under the supervision of an experienced clinician following a well-established clinical protocol. While the clinical recommendation for the vertical distance of mini-implant insertion into the IZC area is 14 to 16 mm measured from the occlusal plane of the maxillary first molar, 9 using the vertical axis of the upper first permanent molar could be a safe method for IZC TADs insertion 15 ( Fig. 2 ), also it is important to note that the optimal height for insertion is higher for male than female. 16

**Fig. 3:**
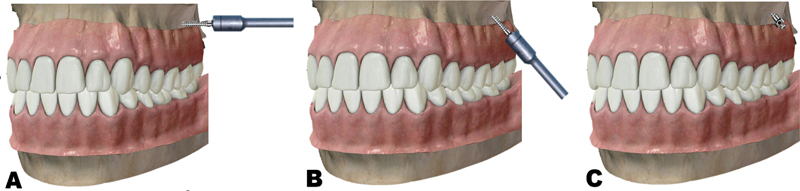
( **A** ) Orientation of the infrazygomatic crest (IZC) temporary anchorage device (TAD) at the start of the installation procedure 90 degrees to the buccal cortical plate. ( **B** ) Change the direction by 55 to 70 degrees after penetration of the buccal cortical plate by approximately 1 mm. ( **C** ) Final position of IZC TAD after insertion.

After ascertaining the insertion point and marking it with a dental probe, a self-drilling mini-implant was placed at 90 degrees to the buccal cortical plate at that point; after a couple of turns to the driver, an initial notch in the bone was created, after which the bone screw driver direction changed by 55 to 70 degrees toward the tooth crown (downward), which helps bypass the roots of the teeth and direct the screw into the IZC area of the maxilla. 17 The mini-implant was screwed until only the head of the screw was visible outside the alveolar mucosa ( Fig. 3 ). Mini-implants were applied for the retraction and distalization mechanics of teeth in the upper arch. The installed mini-implants were loaded with about 227 to 397 g of force per side for an average of 6 months using an elastic power chain (Ormco, Glendora, California, United States). 14 18

### Postoperative Care Instructions

The postoperative care instructions are as follows:

1. Gently brush the miniscrew and use of soft bristle toothbrush.
2. Do not touch miniscrew with the tongue or fingers.
3. Avoid eating hard food during the first 2 days of insertion.
4. Do not tap the miniscrew head with the toothbrush.

## Data Management and Analysis

### Data Collection

#### Postoperative Pain

The patients were asked to record any pain experienced on a visual analog scale (VAS) score sheet at 24 hours and 1 week postplacement 19 (10 = severe pain and 0 = no pain).

#### Primary Stability

For each patient, the primary stability was measured immediately after insertion using EasyCheck (on a scale from 1 to 99) (EasyCheck Genoss Co., Ltd, Jagok-ro, Gangnam-gu, Seoul, Republic of Korea).

**Fig. 4:**
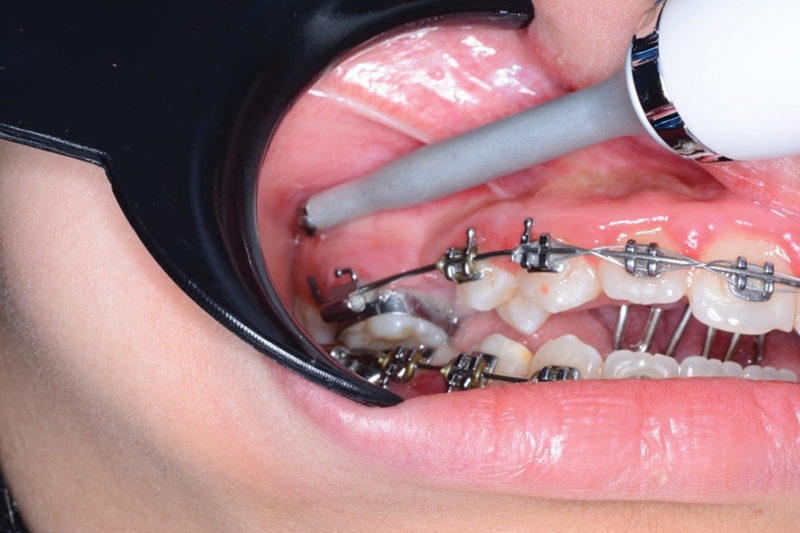
Stability measuring using EasyCheck device where the attack pole directed about 90 degrees to the temporary anchorage device head.

The attack pole was directly connected perpendicularly 90 degrees to the mini-implant head as recommended by the manufacturer ( Fig. 4 ). 20 Stability measurement was applied immediately after TAD insertion since primary stability depends on the mechanical engagement of the mini-implant and bone, and it does not require a period for osseointegration. 21

#### Sinus Perforation

**Fig. 5:**
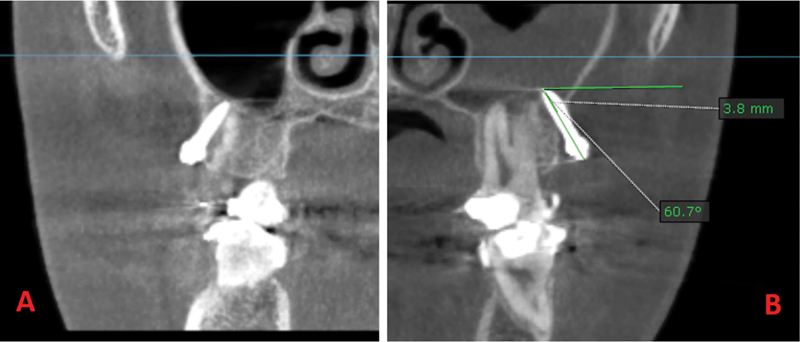
Typical reactions of the maxillary sinus membrane to different penetration depths: ( **A** ) and ( **B** ) are the cone beam computed tomography images obtained immediately after insertion. ( **A** ) There is no penetration while ( **B** ) penetration depth is about 3.8 mm.

Immediately after surgery, a CBCT scan (3D eXam Plus; KaVo Dental, Biberach, Germany) was performed to verify the implant position relative to the adjacent roots and maxillary sinus to evaluate the incidence and degree of root proximity and sinus penetration/perforation, respectively. 10 The OnDemand3D software was used to measure the incidence of penetration and the distance between the distal tip of the mini-implant and the cortical plate of the sinus floor by tracing its long axis. The value was labeled as positive if the mini-implant penetrated the interior wall of the sinus 18 ( Fig. 5 ).

#### Late Stability

The mini-implants' late stability was evaluated 2 months postinsertion using the same device and method for measuring primary stability.

#### Failure Rate

Failure was defined as the mini-implant having to be removed due to looseness or peri-implant inflammation or that it had fallen out after placement. Its stability was reassessed regularly every 3 weeks over 6 months. 22

### Reliability

One calibrated investigator made all measurements. The sinus penetration and mini-implant stability measurements were repeated at 2-week intervals in five randomly selected patients (10 mini-implants). The intraclass correlation coefficient was used to assess intraexaminer reliability for mini-implant stability (0.76), while the kappa test was used to assess intraexaminer reliability for sinus penetration (0.79; Table 1 ). These findings are considered good and reliable for these measurement techniques; therefore, some simple modifications could be made to the technique to increase accuracy.

**Table 1:** Results of reliability tests for penetration and mini-implant stability measurements

Reliability analysis	*N*	Value	Approximate significance
Stability reliability (ICC)	10	0.76	0.003
Sinus penetration reliability (kappa test) test	10	0.79	0.007

Abbreviation: ICC, intraclass correlation coefficient.

### Data Analysis

The data were analyzed using the SPSS software (version 26.0; IBM Corp., Armonk, New York, United States). Since pain perception, success rate, and sinus penetration were nonparametric, they were reported using nonparametric descriptive statistics and compared between groups using Wilcoxon's test. Since the stability measurements were scale data, their normality was assessed using Shapiro–Wilk's test. Primary stability was not normally distributed, so it was compared between groups using Wilcoxon's test. Late stability was normally distributed, so it was compared between the groups using a paired- *t* test.

## Results

The mean primary stability measurement was significantly lower for the 12-mm (44.3) than for the 14-mm (46.5) mini-implants ( *p* < 0.05; Table 2 ). However, the mean late stability measurement did not differ significantly between the 12-mm (36.5) and 14-mm (37.5) mini-implants ( *p* > 0.05; Table 3 ).

**Table 2:** Descriptive and comparative statistics for the primary stability of the two mini-implant lengths (12 mm/14 mm)

	Length	*N*	Descriptive statistic				Comparative statistic	
			Minimum	Maximum	Mean	SD	WSR test	*p* -Value a
Primary stability	12 mm	24	37	60	44.3	5.5	−2.428	0.015
	14 mm	24	39	56	46.5	5.7		

Abbreviations: SD, standard deviation; WSR, Wilcoxon's signed rank.

a *p* -Value < 0.05 is considered as significant.

**Table 3:** Descriptive and comparative statistics for the two lengths (12 mm/14 mm) mini-implant late stability

	Length	*N*	Descriptive statistic				Comparative statistic	
			Minimum	Maximum	Mean	SD	Paired *t* -test	*p* -Value
Late stability	12 mm	24	30	44	36.5	3.7	−1.397	0.176
	14 mm	24	30	47	37.5	4.5		

Abbreviation: SD, standard deviation.

Note: *p* -Value < 0.05 is considered as significant.

Similarly, the sinus penetration rate did not differ significantly between the 12-mm (54.2%) and 14-mm (62.5%) mini-implants ( *p* > 0.05; Table 4 ). Moreover, the success rate did not differ significantly between the 12-mm (79.2%) and 14-mm (83.3%) mini-implants ( *p* > 0.05; Table 5 ).

**Table 4:** Maxillary sinus penetration descriptive and comparative statistics

Length	Sinus penetration	*N*	Descriptive statistic		Comparative statistics	
			Frequency	Percentage	WSR test	*p* -Value
12 mm	No penetration	24	11	45.8%	−0.816	0.414
	Penetration		13	54.2%		
14 mm	No penetration	24	9	37.5%		
	Penetration		15	62.5%		

Abbreviation: WSR, Wilcoxon's signed rank.

Note: *p* -Value < 0.05 is considered as significant.

**Table 5:** Descriptive and comparative statistics for failure rate comparison between the two lengths mini-implants (12 mm/14 mm)

Length	Status	*N*	Descriptive statistic		Comparative statistic	
			Frequency	Percentage	WSR test	*p* -Value
12 mm	Failure	24	5	20.8%	−0.447	0.655
	Success		18	79.2%		
14 mm	Failure	24	4	16.7%		
	Success		20	83.3%		

Abbreviation: WSR, Wilcoxon's signed rank.

Note: *p* -Value < 0.05 is considered as significant.

Regarding pain perception, while most patients experienced pain on the first day, it was greater with the 14 mm than with the 12-mm mini-implants ( Table 6 ). For the 14-mm mini-implants, 16.7% were associated with severe pain and 4.2% with unbearable pain. In contrast, for the 12-mm implants, most were associated with mild to moderate pain, 12.5% with severe pain, and none with unbearable pain. After 1 week, only 4.1% of the 12-mm and 12.5% of the 14-mm mini-implants were associated with mild pain ( Table 7 ).

**Table 6:** Descriptive and comparative statistics for patients' pain perception on the first-day postinsertion

Length	Pain perception	*N*	Descriptive statistic		Comparative statistic	
			Frequency	Percentage	WSR test	*p* -Value
12 mm	No pain	24	1	4.2%	−0.816	0.415
	Mild		10	41.7%		
	Moderate		10	41.7%		
	Severe		3	12.5%		
	Unbearable		0	0		
14 mm	No pain	24	1	4.2%		
	Mild		9	37.5%		
	Moderate		9	37.5%		
	Severe		4	16.7%		
	Unbearable		1	4.2%		

Abbreviation: WSR, Wilcoxon's signed rank.

Note: *p* -Value < 0.05 is considered as significant.

**Table 7:** Descriptive and comparative statistics for patients' pain first-week postinsertion

Length	Pain first week	*N*	Descriptive statistic		Comparative statistic	
			Frequency	Percentage	WSR test	*p* -Value
12 mm	No pain	24	23	95.8%	−1.414	0.157
	Mild		1	4.1%		
	Moderate		0	0%		
	Severe		0	0%		
	Unbearable		0	0%		
14 mm	No pain	24	21	87.5%		
	Mild		3	12.5%		
	Moderate		0	0%		
	Severe		0	0%		
	Unbearable		0	0%		

Abbreviation: WSR, Wilcoxon's signed rank.

Note: *p* -Value < 0.05 is considered as significant.

## Discussion

### TAD Stability in the IZC

Mini-implant stability can be divided into primary (mechanical) and late stability. 23 Long-term dental implant success depends mainly on osseointegration. 24 Orthodontic mini-implant success primarily depends on mechanical stability, so any signs of mini-implant loosening and lack of primary stability within the bone may result in imminent failure of the orthodontic treatment; therefore, stability must be checked early. 25 26 Since primary stability depends on the mechanical engagement between the mini-implant and the bone, it does not require a period of osseointegration. Therefore, primary stability was measured immediately after the insertion of the mini-implants into the IZC area. 21 Many factors affect stability, including bone quantity and quality at the insertion site and mini-implant design, such as length/diameter, thread form/size, pitch, material, and insertion method. 27 28 29

Previous studies have shown that mechanical engagement with surrounding bone is greater with larger screws, producing greater stability. 30 31 Results of this study found that primary stability was significantly lower for 12-mm mini-implants (mean = 44.3) than for 14-mm mini-implants (mean = 46.5; *p* = 0.015). Therefore, the longer mini-implants were more stable. However, while the late stability measurements taken 2 months after mini-implant insertion showed decreased stability for both lengths, the longer mini-implants were still more stable, although the difference was nonsignificant ( *p* = 0.176). This difference may be explained by the longer mini-implants having a greater surface area of engagement with the bone, resulting in more bone contact and, thereby, greater stability. 8

### Sinus Penetration

Hollow spaces around the nose, the maxillary sinuses are the first paranasal sinuses to develop and the largest sinuses in the head. There are two maxillary sinuses in the maxillary bones located in the cheek area next to the nose. They are lined with a membrane called the Schneiderian membrane, which is attached to the interior wall of the maxillary sinus. It is formed from a thin pseudociliated stratified respiratory epithelium layer overlaid by the periosteum layer. It establishes an essential barrier for the defense and protection of the sinus cavity. Its integrity is vital for normal sinus function. 32 This study aimed to measure the incidence of sinus penetration by mini-implants, which was labeled as positive if the mini-implant penetrated the interior wall of the sinus.

Regarding the incidence of sinus penetration, Chang et al 10 found that 48.0% of TADs inserted into the IZC perforated the maxillary sinus with a mean depth of 3.23 mm. Jia et al 18 reported a higher penetration incidence of 78.3%. This study showed that the penetration rate was higher with 14-mm TADs (62.5%) than with 12-mm TADs (54.2%), although the difference was nonsignificant.

Reiser et al reported that the sinus membrane became elevated when mini-implants penetrated <2 mm into the maxillary sinus, which enhanced healing since it assists the formation of a blood clot that provides a scaffold for the formation of bone in this region. However, the Schneiderian membrane could be perforated if the mini-implant extends >2 mm into the maxillary sinus, which may result in the discharge of bone fragments inside the maxillary sinus, compromising its healing ability and increasing the risk for sinusitis. 32

Jia et al 18 stated that a penetration depth of <1 mm is recommended for IZC mini-implant anchorage to enhance primary stability. In contrast, Chang et al 10 claimed that perforation by TADs may be unnecessary, with a mean of 3.23 mm resulting in a 21.3% decrease in insertion torque.

Based on the previous studies, it can be concluded that it is better to avoid sinus penetration, but if it happens, keeping it <2 mm may minimally affect the prognosis. The finding of the present study showed that while the longer mini-implants had higher sinus penetration rates and were more likely to go deeper into the sinus (> 2 mm), increasing the risk for side effects such as sinusitis, however, the failure rate of both mini-implants lengths (12 and 14 mm) did not differ significantly as described later.

### Failure Rate

Mini-implants can be considered successful if they are maintained inside the bone until the treatment goals are achieved or their planned removal, whereas mini-implants are considered to have failed if they have severe clinical mobility and cannot act as a stable anchor, necessitating their replacement or removal. 22 Their loss within less than 6 months after placement, the minimal interval for anchorage to retract the maxilla, is also considered a failure. 30 33 Many factors can lead to mini-implant failure, such as their loosening due to inflammation around the insertion site, overloading, cortical bone thickness and mineral density, screw design, and root proximity. 34 35 36

In the present study, TAD was considered failed if it needed to be removed before treatment goals were achieved due to mini-implant fracture, uncontrollable soft tissue inflammation, severe mobility, and/or host factors (root damage) or if it had fallen out after placement. The failure rate for the mini-implants was checked over 6 months.

Previous studies have reported differing success and failure rates for mini-implants inserted into the IZC region. Jia et al 18 reported an overall success rate of 96.7% for mini-implants inserted into the IZC area. Similarly, Chang et al 14 reported an overall success rate of 93.7% for mini-implants, which is considered clinically high and very optimistic. However, Gill et al 13 reported a failure rate of 28.1%. Similarly, Uribe et al 22 reported a failure rate of 21.8% for mini-implants placed in the IZC, which seems lower than those in the other studies.

The result of this study elicited success rates of 79.2% for the 12-mm mini-implants and 83.3% for the 14-mm mini-implants, although the difference was nonsignificant ( *p* = 1.000). Although lower than those of Jia et al and Chang et al, these values of success rates are still within an acceptable range and clinically applicable. The differences in success/failure rates between the present study and the previous studies could be related to several factors, such as the study design, study sample, patient characteristics, and the specific criteria used to define success or failure.

Factors such as sex, age, mini-implant length (12/14 mm), occlusogingival position, force application method, and insertion angle may not be significantly related to lower or higher odds of mini-implant failure. 13 37 Nonetheless, the present finding may agree with Gill et al, 13 who found no significant difference in failure rate between these two mini-implant sizes. 13

Although failure rates do not appear to differ significantly for both longer and shorter mini-implants, the longer mini-implants undoubtedly may still have disadvantages regarding possible side effects. Indeed, they are more likely to damage adjacent structures. Therefore, a shorter mini-implant should be preferred over longer mini-implants whenever possible.

### Pain Perception

Sarul et al 31 used two mini-implant sizes in the BS area, showing that smaller mini-implants were significantly better tolerated by patients than the larger mini-implants. Kuroda et al 38 reported that about 60% of patients given larger mini-implants experienced pain on the third-day postinsertion.

The current study assessed pain perception using a VAS, showing that while most patients still experienced pain on the first day, it was more severe on the side treated with the longer mini-implants. Moreover, while pain had noticeably reduced after 1 week, some patients still felt mild pain or discomfort, which was reported for 4.1% of the 12-mm mini-implants versus 12.5% for the 14-mm mini-implants.

These findings agree with Sarul et al and Kuroda et al, who reported that longer mini-implants produced more discomfort, which was clinically important but not significantly different. One possible cause of the difference in pain perception could be the difference in length, with longer mini-implants going deeper into the sinus and having a higher likelihood of damaging the Schneiderian membrane, potentially resulting in the discharge of bone fragments inside the maxillary sinus, compromising its ability to heal and increasing the risk for sinusitis. 32 However, the major concern for postoperative pain is individual-specific and subjective.

## Conclusion

1. There could be a correlation between mini-implant length and sinus penetration.
2. The mini-implant length may have an association with pain perception at the first-week postinsertion.
3. There could be no significant correlation and the mini-implant length and failure rate.
4. Shorter mini-implant may be as efficient as a larger one and could be safer.

## Footnotes

a Title page
